# Effects of an 8-week self-compassion-based mental preparation program on mood states and 3,000 m performance in cadet cross-country athletes

**DOI:** 10.3389/fpsyg.2026.1904853

**Published:** 2026-09-16

**Authors:** Najha Bouallagui, Chiraz Goumni, Achraf Hammami, Adel Driss, Riadh Khalifa, Raouf Hammami, Imen Ben Amar

**Affiliations:** 1 Higher Institute of Sport and Physical Education of Ksar-Said, University of Manouba, Manouba, Tunisia; 2 Research Laboratory (LR23JS01) Sport Performance, Health & Society, Higher Institute of Sport and Physical Education of Ksar-Said, University of Manouba, Tunis, Tunisia; 3 Department of Physiology, Morehouse School of Medicine, Atlanta, GA, United States; 4 Tunisian Research Laboratory ‘Sports Performance Optimization', National Center of Medicine and Science in Sports (CNMSS), Tunis, Tunisia

**Keywords:** cadet athletes, cross-country running, endurance performance, mood states, psychological skills training, self-compassion

## Abstract

**Background:**

Mental preparation is increasingly recognized as a key factor in optimizing both psychological and physical performance in athletes; however, its specific effects on mood states and endurance performance in cross-country runners remain insufficiently investigated.

**Objective:**

This study examined the effects of an 8-week mental preparation program on mood states and 3,000 m performance in cross-country athletes compared with a control group, with a specific focus on self-compassion-based training.

**Methods:**

Thirty-two male cross-country athletes (16 ± years old; 3–5 years of experience) were allocated to an experimental group (*n* = 17) or a control group (*n* = 15). The experimental group completed an 8-week self-compassion-based mental preparation program, while the control group continued regular training. Pre- and post-intervention assessments included the Profile of Mood States (POMS), the Self-Compassion Scale (SCS), and a 3,000 m performance test. Data were analyzed using repeated-measures ANOVA to examine Group × Time interactions.

**Results:**

No significant baseline differences were observed between groups. Significant Group × Time interactions were found for all mood state dimensions (*p* < 0.001), with the experimental group showing greater reductions in anxiety–tension, anger–hostility, confusion–perplexity, depression–dejection, and fatigue–inertia, and greater increases in vigor–activity and interpersonal relationships compared with controls. Significant improvements were also observed in all self-compassion dimensions (*p* < 0.001), with increases in self-kindness, common humanity, and mindfulness, and decreases in self-judgment, isolation, and over-identification in the experimental group. For physical performance, a significant Group × Time interaction was found for the 3,000 m test (F(1,30) = 7.24, *p* = 0.049, η*p*^2^ = 0.12), indicating greater performance improvement in the experimental group (9:55 ± 0:34 to 9:46 ± 0:28 min) compared with controls (9:48 ± 0:29 to 9:49 ± 0:34 min).

**Conclusion:**

An 8-week self-compassion-based mental preparation program was associated with improved psychological wellbeing and a small, modest improvement in endurance performance in young cross-country athletes.

## 1 Introduction

Athletes are regularly exposed to a range of stressors, including performance pressure, setbacks, injuries, and competitive failure. Although physical preparation is fundamental to athletic success, psychological resources also play a critical role in determining how athletes respond to these challenges and maintain optimal performance (Vallerand, 2015). Indeed, psychological skills such as emotional regulation, attentional control, and goal setting have consistently been associated with effective coping and successful performance in sport (Krane and Williams, 2006). Consequently, sport psychology has increasingly focused on identifying factors that promote resilience, wellbeing, and adaptive functioning in athletes.

One factor that has received growing attention is self-compassion. According to (Neff 2003a,b), self-compassion involves responding to personal difficulties with self-kindness rather than self-judgment, recognizing that imperfection and suffering are shared aspects of the human experience, and maintaining a balanced awareness of distressing thoughts and emotions. In contrast to self-criticism, which is prevalent among athletes striving to meet high performance standards, self-compassion may provide a more adaptive way of responding to mistakes, failures, and setbacks. By fostering acceptance and understanding rather than harsh self-evaluation, self-compassion may help athletes regulate negative emotions while maintaining motivation and engagement in their sport.

A substantial body of evidence indicates that self-compassion is associated with psychological wellbeing. Individuals with higher levels of self-compassion typically report lower levels of anxiety, depression, and negative affect, as well as greater emotional resilience and life satisfaction (Barnard and Curry, 2011; Neff, 2011). Within sport settings, self-compassion has been linked to adaptive coping, reduced fear of failure, lower self-criticism, and more constructive responses to performance setbacks (Mosewich et al., 2013; Ceccarelli et al., 2019). Furthermore, recent research suggests that self-compassion serves as an important emotional resource that enables athletes to navigate the demands of competitive sport while preserving psychological wellbeing (Zhang et al., 2023). These findings are consistent with broader psychological research demonstrating that self-compassion reduces maladaptive responses to adversity, such as rumination, self-blame, and catastrophizing (Leary, 2007; Neff and Vonk, 2009).

Recent advances in sport psychology have increasingly emphasized the value of third-wave psychological approaches, particularly those incorporating self-compassion, mindfulness, acceptance, and psychological flexibility. Emerging intervention-based research provides encouraging evidence for the application of these approaches within athletic populations. For instance, the RESET intervention, adapted from the mindful self-compassion program, was associated with increased self-compassion and reductions in self-criticism and fear of self-compassion among student-athletes, supporting the feasibility of integrating self-compassion into sport settings (Kuchar et al., 2023). Similarly, (Reis et al. 2023) demonstrated the feasibility and acceptability of a self-compassion intervention among competitive male athletes, suggesting its potential to promote more adaptive responses to challenging sport-related experiences. These findings align with the broader development of mindfulness- and acceptance-based approaches, in which psychological flexibility is regarded as an important process underlying adaptive psychological functioning (Macri and Rogge, 2024). This perspective may be particularly relevant during adolescence, as recent evidence highlights the importance of flexible emotion regulation during this developmental period, while empirical research in adolescent populations remains relatively limited (Haag et al., 2024).

Despite growing interest in self-compassion within sport psychology, several gaps remain. First, much of the available evidence is based on correlational research, limiting conclusions about the causal effects of self-compassion on athlete functioning. Although intervention studies have shown that self-compassion can be cultivated and may improve psychological outcomes such as self-criticism, emotional wellbeing, and coping (Killham et al., 2018; Mosewich et al., 2019), relatively few studies have examined its effects on competitive athletes. Second, the relationship between self-compassion and athletic performance remains unclear. While self-compassion appears to facilitate adaptive emotional responses to failure and stress (Ceccarelli et al., 2019), evidence for direct improvements in objective performance outcomes is limited. This distinction is important because psychological interventions may enhance performance indirectly through improved emotional regulation, recovery, and persistence rather than through immediate performance gains. Third, existing intervention research has focused primarily on adult or collegiate athletes, leaving adolescent athletes largely underrepresented.

The potential influence of self-compassion on mood states warrants particular attention. Mood has long been recognized as an important determinant of athletic performance and psychological functioning (Lane and Terry, 2000; Lane et al., 2012). Negative mood states, including tension, depression, anger, and confusion, have been associated with poorer performance and reduced wellbeing, whereas positive mood states are generally linked to more favorable outcomes. Given that self-compassion promotes adaptive emotional regulation and reduces negative self-evaluative processes, it may be especially effective in improving athletes' mood profiles. This possibility may be particularly relevant for endurance athletes, who frequently encounter fatigue, discomfort, and performance-related setbacks that require sustained psychological resilience.

Therefore, the purpose of the present study was to investigate the effects of a self-compassion intervention on mood states and endurance performance among cadet distance runners. Based on previous evidence linking self-compassion to emotional wellbeing, adaptive coping, and resilience in sport (Mosewich et al., 2013; Killham et al., 2018; Ceccarelli et al., 2019; Zhang et al., 2023), it was hypothesized that athletes receiving the self-compassion intervention would demonstrate greater improvements in mood states, reflected by reduced negative affect and enhanced positive affect, compared with a no-intervention control group. Furthermore, because self-compassion may facilitate more adaptive responses to training and competitive stressors (Ceccarelli et al., 2019), it was expected that athletes in the intervention group would exhibit greater improvements in 3,000-m running performance. Given the limited empirical evidence regarding direct performance effects, this latter hypothesis was considered exploratory.

## 2 Methods

### 2.1 Overview and design

A quasi-experimental pretest–posttest control-group design was employed to examine the effects of a self-compassion intervention on mood states and endurance performance. Participants were assigned to either an experimental group (EG) or a control group (CG) according to pre-existing training group membership; therefore, random allocation was not feasible. The EG participated in a structured self-compassion intervention adapted from the mindful self-compassion program developed by (Neff and Germer 2013), whereas the CG continued its usual training routine and did not receive any psychological intervention during the study period. Outcome measures were collected before and after the intervention in both groups. A flow diagram illustrating participant allocation, intervention delivery, and assessment procedures is presented in Figure 1.

**Figure 1:**
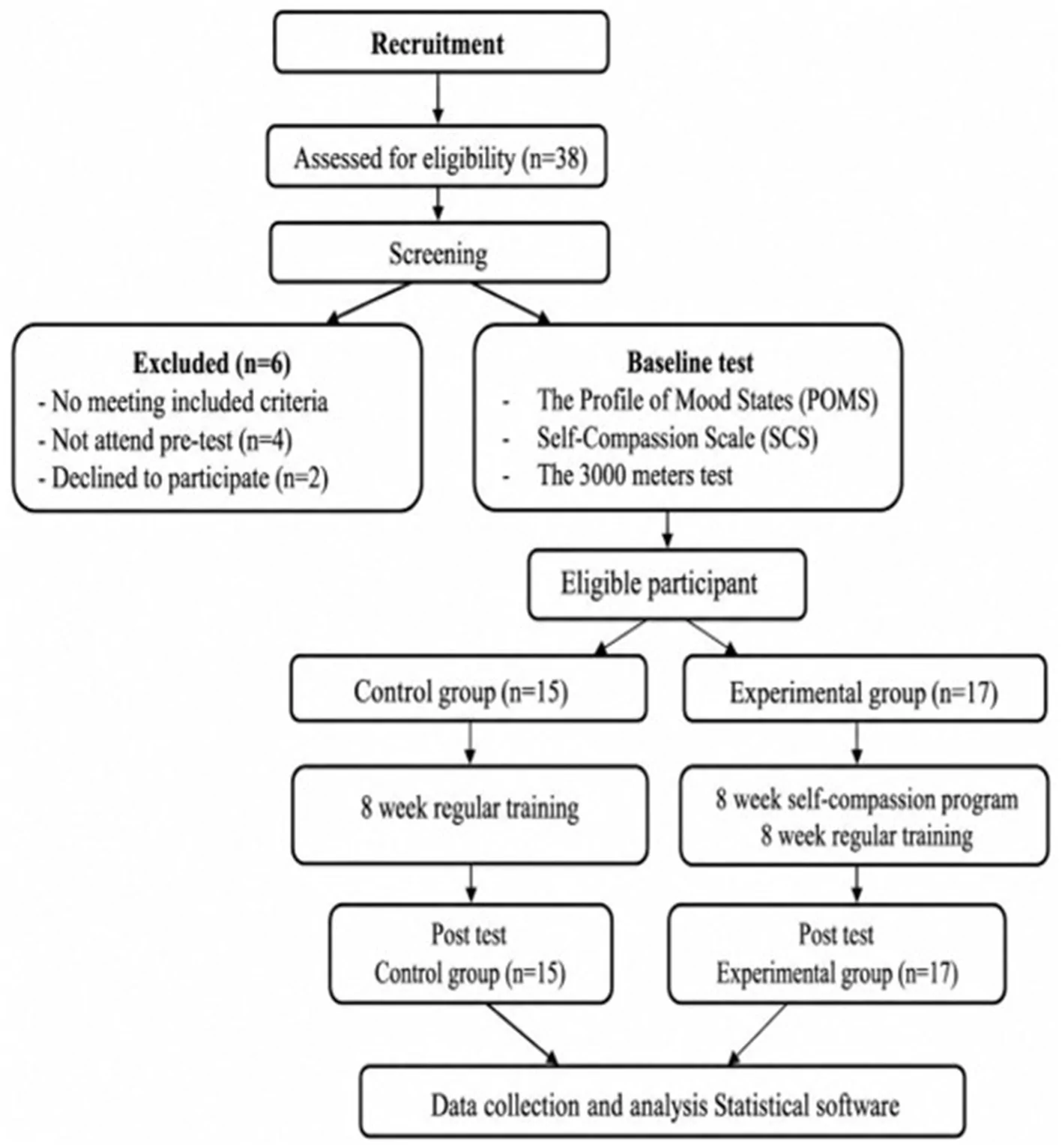
Flow diagram of participants' training schedule through the study.

### 2.2 Participants

An *a priori* power analysis was conducted using G^*^Power 3.1 (Faul et al., 2009) to determine the minimum required sample size. Assuming a medium effect size (*f* = 0.25), an alpha level of 0.05, and a statistical power of 0.80, the analysis indicated that at least 28 participants were required. A total of 38 athletes were initially recruited; following screening and completion of the study protocol, the final sample consisted of 32 male cadet cross-country runners. Participants were 16.0 ± 0.8 years old and had a minimum of three years of competitive running experience. All athletes competed at the national level and trained within the same athletics club, ensuring comparable training conditions. Weekly training volume ranged between 8 and 12 h of structured training. Baseline anthropometric characteristics are presented in Table 1. At study entry, participants in the control group (CG; *n* = 15) and experimental group (EG; *n* = 17) showed similar body mass (CG: 62.50 ± 1.22 kg; EG: 61.57 ± 1.50 kg), height (CG: 168.14 ± 3.35 cm; EG: 167.29 ± 3.24 cm), and body mass index (CG: 21.98 ± 0.89 kg/m^2^; EG: 22.01 ± 0.73 kg/m^2^), indicating comparable baseline physical characteristics. Eligibility criteria included active participation in competitive cross-country running and the absence of psychological, neurological, musculoskeletal, or orthopedic disorders during the 6 months preceding and throughout the study. Recruitment of participants took place during the pre-competitive period of the sports season. Allocation to groups was made according to membership in pre-existing training groups, after obtaining the written consent of the participants and the agreement of their parents or legal guardians. Adherence to the program was strictly controlled in the experimental group: any absence from a session resulted in the exclusion of the participant, this rule aimed at preserving the continuity of the intervention over a limited period of 8 weeks, All participants completed the study protocol. The study followed a quasi-experimental design, and baseline comparisons confirmed no meaningful differences between groups in anthropometric variables. The study was conducted in accordance with the Declaration of Helsinki and was approved by the Local Ethics Committee of the National Center of Medicine and Science of Sports of Tunis (CNMSS-LR09SEP01). Written informed consent was obtained from all participants and their parents or legal guardians prior to participation.

**Table 1:** Weekly structure of the 8-week self-compassion intervention program.

Week	focus	Psychological objective	Main activities
Week 1	Introduction to self-compassion	Understanding self-compassion and its role in sport performance	Psychoeducation on Neff's model; discussion on self-criticism in sport; baseline reflection on personal responses to failure
Week 2	Awareness of self-critical thoughts	Identifying negative self-talk in training and competition	Self-monitoring of self-critical thoughts; mindfulness breathing (5–10 min); reflection on emotional reactions to mistakes
Week 3	Developing self-kindness	Replacing self-criticism with supportive self-talk	Compassionate letter-writing after perceived failure; cognitive reframing of negative thoughts
Week 4	Common humanity	Normalizing failure and setbacks	Group discussion of shared sport difficulties; reflection on elite athletes' setbacks; perspective-taking exercise (“what would I say to a teammate?”)
Week 5	Mindfulness and emotional regulation	Improving emotional acceptance and control	Guided mindfulness practice; acceptance of emotions; introduction of brief reset routines (breathing + grounding)
Week 6	Self-compassion and motivation	Promoting adaptive achievement motivation	Discussion on self-criticism vs. self-improvement; identification of personal sport values; linking goals to personal growth
Week 7	Application to competition	Transfer of skills to competitive contexts	Simulation of competition stress; visualization with compassionate self-talk; development of individualized coping plan
Week 8	Integration and maintenance	Consolidation of skills and long-term application	Post-intervention assessment; group reflection; creation of personal maintenance plan for future training and competition

### 2.3 Procedures

A quasi-experimental pretest–posttest design was employed. Data were collected at two time points (pre- and post-intervention) under standardized conditions. The experimental group completed an 8-week self-compassion intervention, whereas the control group continued their usual training routine without psychological intervention. Both groups underwent identical assessment procedures at baseline and post-intervention. At baseline, all participants completed the POMS and SCS questionnaires and performed the 3,000-m test under standardized conditions. The intervention then took place over 8 weeks. Post-intervention assessments replicated baseline procedures using identical testing conditions, timing, and protocols to ensure comparability across measurement occasions.

### 2.4 Anthropometric and performance measures

Height (cm) and body mass (kg) were measured using standard anthropometric procedures. Body mass index (BMI) was calculated as body mass (kg) divided by height (m^2^).

Endurance performance was assessed using a 3,000-m cross-country running test conducted on a standardized outdoor course with natural terrain features to reflect competitive conditions. Participants were instructed to complete the distance in the shortest possible time. Performance time was recorded using electronic timing systems to ensure accuracy and reduce measurement error.

All testing sessions were conducted under comparable environmental conditions (temperature: 18 °C−22 °C; humidity: 50%−65%; wind speed ≤ 2 m/s). Participants completed a standardized warm-up consisting of 10 min of light jogging, dynamic stretching, and progressive accelerations. Testing sessions were scheduled at least 48 h apart from strenuous training to minimize fatigue effects. Anthropometric assessments were conducted between 09:00 and 11:00, while performance testing took place at approximately 16:00 to ensure standardized timing across sessions.

### 2.5 Psychological measures

Mood states were assessed using the French version of the Profile of Mood States (POMS; Mcnair et al., 1971; Cayrou et al., 2003). The questionnaire consists of 65 items rated on a 5-point Likert scale (0 = not at all to 4 = extremely) and assesses seven dimensions: tension-anxiety, depression, anger, vigor, fatigue, confusion, and interpersonal relationships, as well as a total mood disturbance score. The POMS has demonstrated McDonald's ω coefficients of 0.75 for Anxiety-Tension, 0.72 for Depression-Descouragement, 0.86 for Anger-Hostility, 0.72 for Vigor-Activity, 0.76 for Fatigue-Inertia, 0.82 for Confusion-Perplexity, Interpersonal relationship 0.81 and 0.80 for the total scale.

Self-compassion was measured using the Self-Compassion Scale (SCS; Neff, 2003a,b). The scale comprises 26 items rated on a 5-point Likert scale (1 = almost never to 5 = almost always) and includes six subscales: self-kindness, self-judgment, common humanity, isolation, mindfulness, and over-identification. A total self-compassion score was computed, with higher scores indicating greater self-compassion. The SCS has demonstrated good psychometric properties and has been validated in sport populations (Mosewich et al., 2013). In the present study, McDonald's ω coefficients were 0.81for Self-Kindness, 0.70 for Self-Judgment, 0.74 for Common Humanity, 0.76 for Isolation, 0.80 for Mindfulness,0.73 for Over-Identification, and 0.82 for the total scale, indicating acceptable to good internal consistency across the Self-Compassion Scale dimensions.

### 2.6 Self-compassion intervention

The experimental group participated in an 8-week structured self-compassion intervention adapted from Neff's self-compassion framework and the Mindful Self-Compassion program (Neff, 2003a,b; Neff and Germer, 2013). The program consisted of one weekly session of 60–75 mins, led by two doctoral students in Sports Science (STAPS), trained in the self-compassion approach. Each session followed a standardized structure: it began with a breathing exercise designed to create a positive and supportive atmosphere, continued with a review of the previous session in the form of constructive and non-judgmental feedback, and then addressed the specific content planned for the week. No homework or exercises were assigned between sessions. Participant attendance was rigorously monitored, with any absence resulting in the participant's exclusion from the experimental group. Before the intervention began, informed consent was obtained from each participant and their legal representative. Finally, this protocol is not a direct replication of the Mindful Self-Compassion program (Neff and Germer, 2013), but an adaptation designed to meet the requirements of the competitive sports context and the characteristics of the cadet athlete population (Table 1). Each session combined psychoeducation, experiential exercises, reflection tasks, and sport-specific applications designed to help athletes develop self-kindness, mindfulness, and a sense of common humanity in response to training and competition-related stressors. The intervention progressed from an initial introduction to self-compassion and awareness of self-critical thoughts, toward the development of self-kindness strategies, normalization of failure experiences, and mindfulness-based emotional regulation skills. Subsequent sessions focused on enhancing adaptive motivation, applying self-compassion strategies in simulated competitive contexts, and developing individualized coping plans for use in training and competition. The final session emphasized consolidation of skills and the formulation of a maintenance plan to support long-term application. Throughout the program, athletes were encouraged to apply self-compassion principles to real-life sport situations, particularly in response to mistakes, setbacks, and performance pressure.

### 2.7 Statistical analysis

Data analysis was performed using SPSS software (version 26.0; IBM Corp., Armonk, NY, USA). Descriptive statistics (mean ± standard deviation) were calculated for all variables. The normality of data distribution was assessed using the Shapiro–Wilk test. Baseline differences between groups were examined using one-way ANOVA to confirm group equivalence prior to the intervention. To evaluate the effects of the intervention, a 2 (Group: Experimental vs. Control) × 2 (Time: Pre-test vs. Post-test) mixed-design repeated-measures analysis of variance (ANOVA) was conducted, with effect sizes reported using partial eta squared (η*p*^2^). To further account for potential baseline imbalances, between-group differences at post-intervention were also examined using analysis of covariance (ANCOVA), with baseline scores entered as covariates; adjusted means, 95% confidence intervals, and between-group mean differences are reported. Internal consistency of the SCS subscales was assessed using McDonald's omega (ω), with values considered acceptable at ω ≥ 0.70. Statistical significance was set at *p* < 0.05.

## 3 Results

### 3.1 Baseline characteristics and assumption testing

No significant baseline differences were observed between the control and experimental groups in body mass, height, or body mass index (BMI); (Table 2). The data were normally distributed, and the assumption of homogeneity of variance was satisfied (all *p* > 0.05).

**Table 2:** Characteristics of participants in a control and experimental group.

Characteristics	Control group		Experimental group	
	Baseline	Post	Baseline	Post
Body mass (kg)	62.50 ± 1.22	62.64 ± 0.84	61.57 ± 1.50	62.36 ± 1.01
Height (cm)	168.14 ± 3.35	168.14 ± 3.35	167.29 ± 3.24	167.29 ± 3.24
Body mass index (kg/m^2^)	21.98 ± 0.89	22.19 ± 0.90	22.01 ± 0.73	22.29± 0.93

### 3.2 Mood states outcomes

Pre- and post-intervention results are presented in Table 3. Repeated measures ANOVA revealed significant Group × Time interactions for all mood state variables, indicating greater improvements in the experimental group compared with the control group (all *p* < 0.001). Specifically, significant increases were observed in vigor–activity and interpersonal relationships, whereas significant decreases were found in anxiety–tension, anger–hostility, confusion–perplexity, depression–dejection, and fatigue–inertia (Figures 2–4).

**Table 3:** Comparison of profile mood state dimension, self-compassion dimension and physical performance between pre- and post-intervention in a control and intervention group.

Variable	Group	Pre	Post	Δ (%)	*p-*value			*ηp* ^2^		
					Group	Time	G×T	Group	Time	G×T
Anxiety-tension	Control	31.07 ± 2.08	30.80 ± 2.32	−1 %	0.001^*^	0.001^*^	0.001^*^	0.387	0.807	0.778
	Experimental	31.20 ± 2.07	25.00 ± 1.51	−20 %						
Anger-hostility	Control	26.33 ± 2.35	26.93 ± 2.40	2 %	0.835	0.001^*^	0.001^*^	0.001	0.361	0.535
	Experimental	28.35 ± 3.98	25.58 ± 3.75	−10 %						
Confusion-perplexity	Control	22.13 ± 1.15	21.63 ± 1.41	−2 %	0.001^*^	0.001^*^	0.001^*^	0.341	0.593	0.450
	Experimental	21.81 ± 1.33	18.31 ± 2.15	−16 %						
Depression-discouragement	Control	51.65 ± 2.31	50.50 ± 2.25	−2 %	0.001^*^	0.001^*^	0.001^*^	0.378	0.846	0.797
	Experimental	53.44 ± 2.13	40.13 ± 5.23	−25 %						
Fatigue-inertia	Control	23.69 ± 1.62	22.93 ± 1.88	−3 %	0.001^*^	0.001^*^	0.001^*^	0.703	0.895	0.852
	Experimental	23.06 ± 1.65	15.31 ± 1.01	−33 %						
Vigor-activity	Control	21.06 ± 1.24	20.56 ± 1.31	−2 %	0.001^*^	0.001^*^	0.001^*^	0.549	0.714	0.771
	Experimental	20.50 ± 2.07	27.25 ± 2.21	34 %						
Interpersonal relationship	Control	19.93 ± 1.09	19.93 ± 1.09	0 %	0.001^*^	0.001^*^	0.001^*^	0.515	0.693	0.693
	Experimental	19.41 ± 1.50	25.58 ± 2.45	35 %						
Global score	Control	133.80 ± 4.42	132.25 ± 5.86	−1 %	0.001^*^	0.001^*^	0.001^*^	0.546	0.860	0.839
	Experimental	137.24 ± 7.05	99.05 ± 12.25	−29 %						
Self-judgment	Control	9.87 ± 0.51	9.80 ± 1.20	0 %	0.001^*^	0.001^*^	0.001^*^	0.343	0.459	0.482
	Experimental	10.18 ± 1.01	7.35 ± 1.36	−46 %						
Over-identification	Control	8.13 ± 0.83	8.20 ±1.14	2 %	0.001^*^	0.001^*^	0.001^*^	0.408	0.504	0.474
	Experimental	8.18 ± 0.63	6.00 ± 1.00	−40 %						
Isolation	Control	7.73 ± 1.43	7.60 ± 0.98	1 %	0.351	0.004^*^	0.001^*^	0.029	0.240	0.293
	Experimental	8.29 ± 1.10	6.35 ±1.61	−42 %						
Mindfulness	Control	15.07 ± 1.10	15.53 ±1.40	3 %	0.031^*^	0.067	0.002^*^	0.146	0.108	0.271
	Experimental	15.24 ± 1.43	16.94 ± 1.39	10 %						
Common humanity	Control	16.53 ± 1.12	15.73 ± 1.03	−3 %	0.009^*^	0.001^*^	0.004^*^	0.204	0.623	0.243
	Experimental	16.00 ± 0.93	18.06 ± 1.19	11 %						
Self-kindness	Control	19.87 ± 1.59	19.60 ± 1.45	0 %	0.009^*^	0.001^*^	0.004^*^	0.206	0.335	0.250
	Experimental	19.53 ± 1.41	22.12 ± 1.57	12 %						
3000 m performance (min: sec)	Control	9:48 ± 0:29	9:49 ± 0:4	0 %	0.859	0.012^*^	0.049^*^	0.001^*^	0.121	0.120^*^
	Experimental	9:55 ± 0:34	9:46 ± 0.28	−1 %						

^*^Mean differences were significant at *p* < 0.05; Δ delta change; η^2^*p*=partial eta squared (effect size).

**Figure 2:**
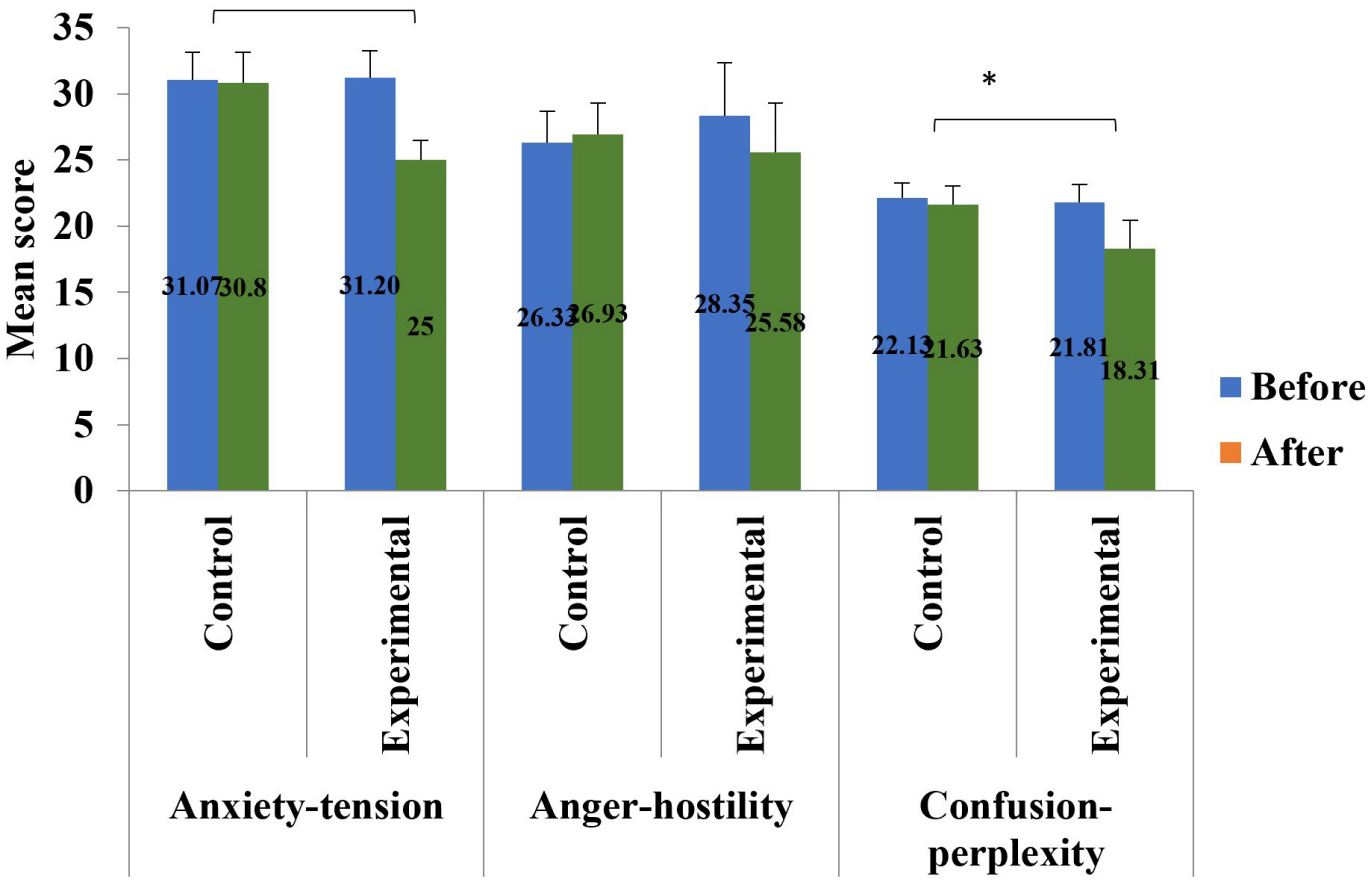
Mean (±SD) profile of mood states (POMS) scores for anxiety–tension, anger–hostility, and confusion–perplexity in the control and experimental groups at baseline and post-intervention. ^*^ Significant differences between pre- and post-intervention are indicated by ^*^p < 0.05.

**Figure 3:**
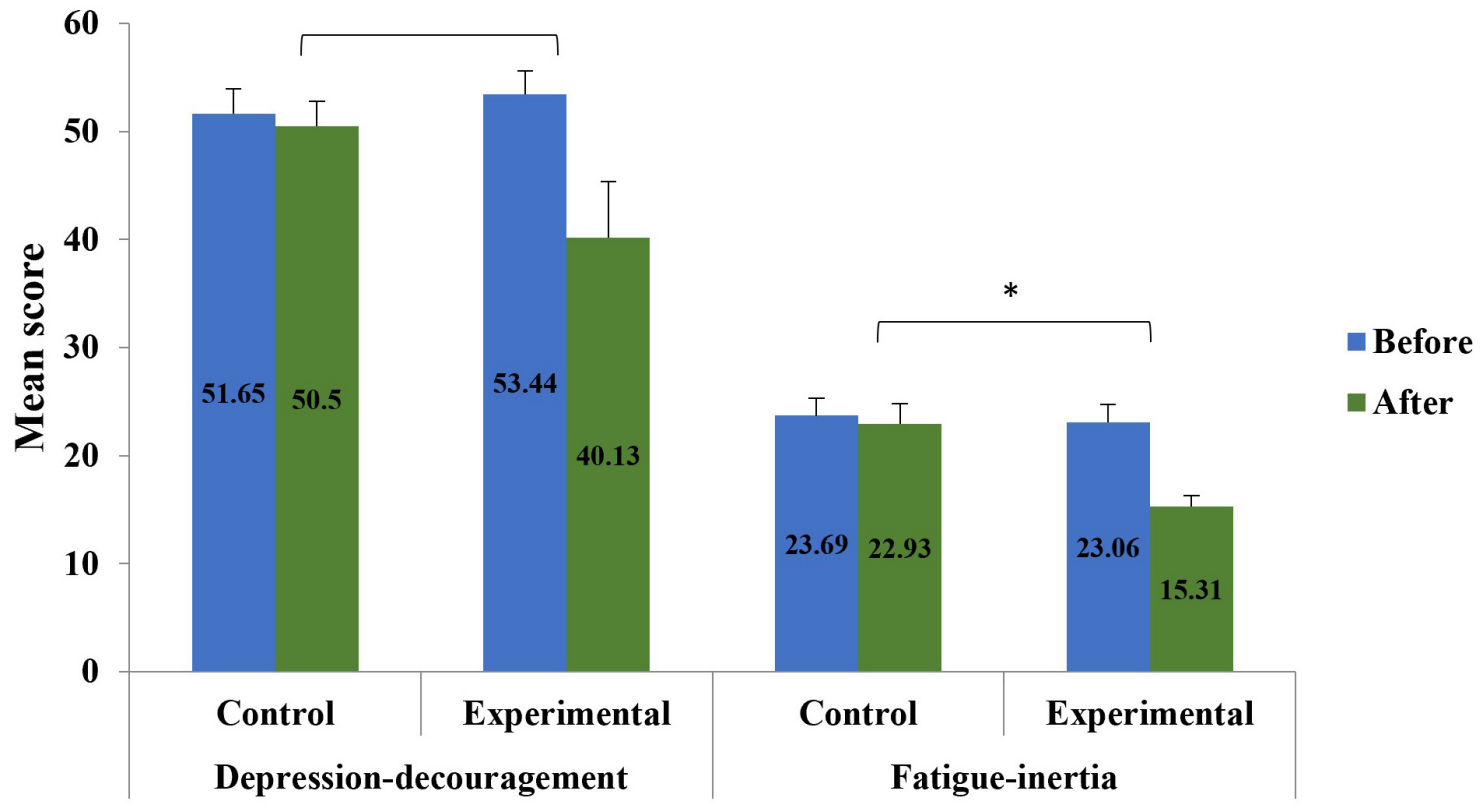
Mean (±SD) profile of mood states (POMS) scores for depression–discouragement and fatigue–inertia in the control and experimental groups at baseline and post-intervention. ^*^ Significant differences between pre- and post-intervention are indicated by *p* < 0.05.

**Figure 4:**
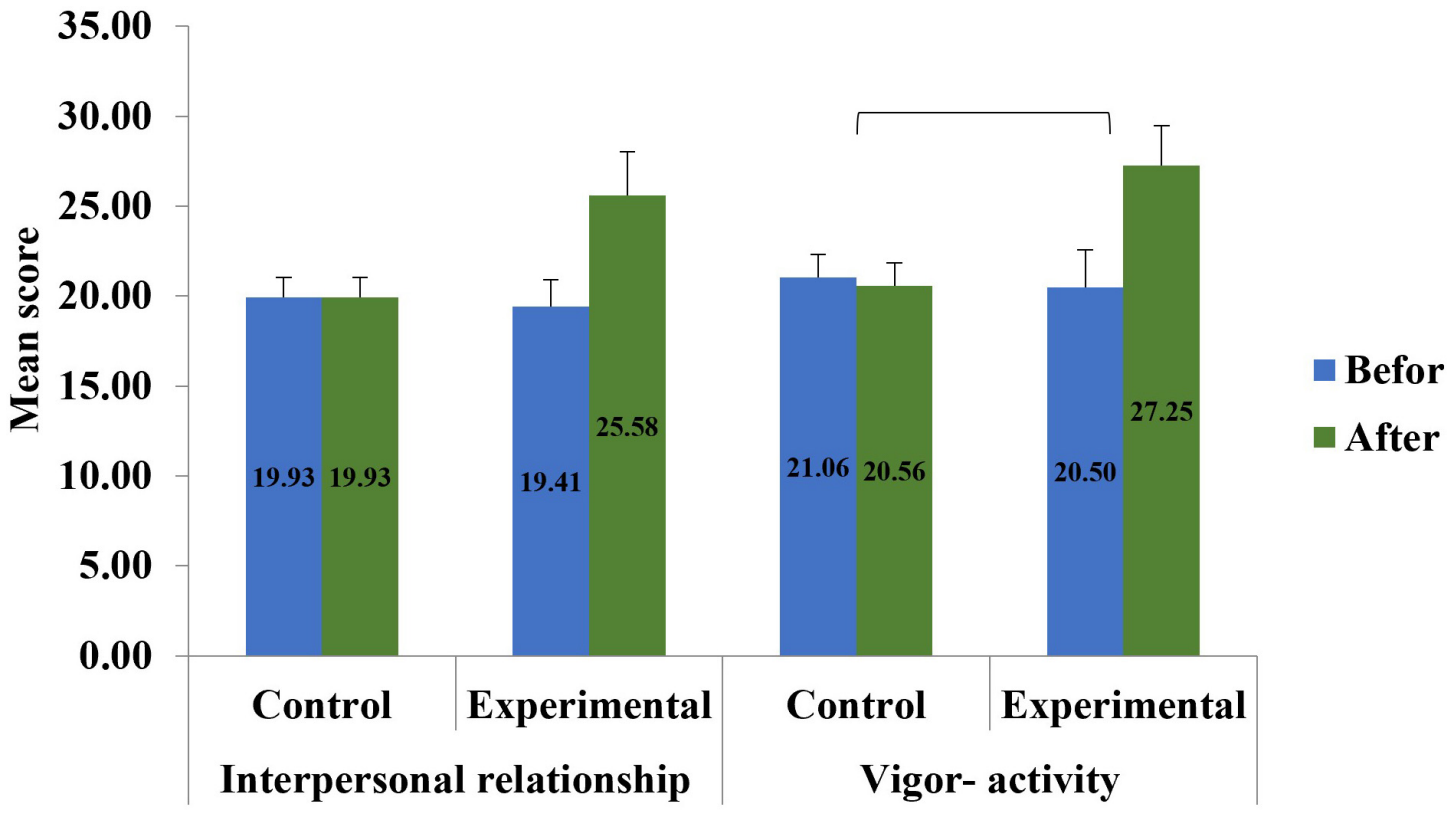
Mean (±SD) profile of mood states (POMS) scores for vigor–activity and interpersonal relationships in the control and experimental groups at baseline and post-intervention. ^*^ Significant differences between pre- and post-intervention are indicated by *p* < 0.05.

### 3.3 Self-compassion outcomes

Significant Group × Time interactions were observed for all self-compassion dimensions, including self-kindness, common humanity, and mindfulness (all *p* < 0.001). The experimental group showed greater improvements following the intervention, alongside significant reductions in self-judgment, isolation, and over-identification (Figures 5, 6). This improvement in SCS scores confirms the fidelity of the intervention and serves as a manipulation check.

**Figure 5:**
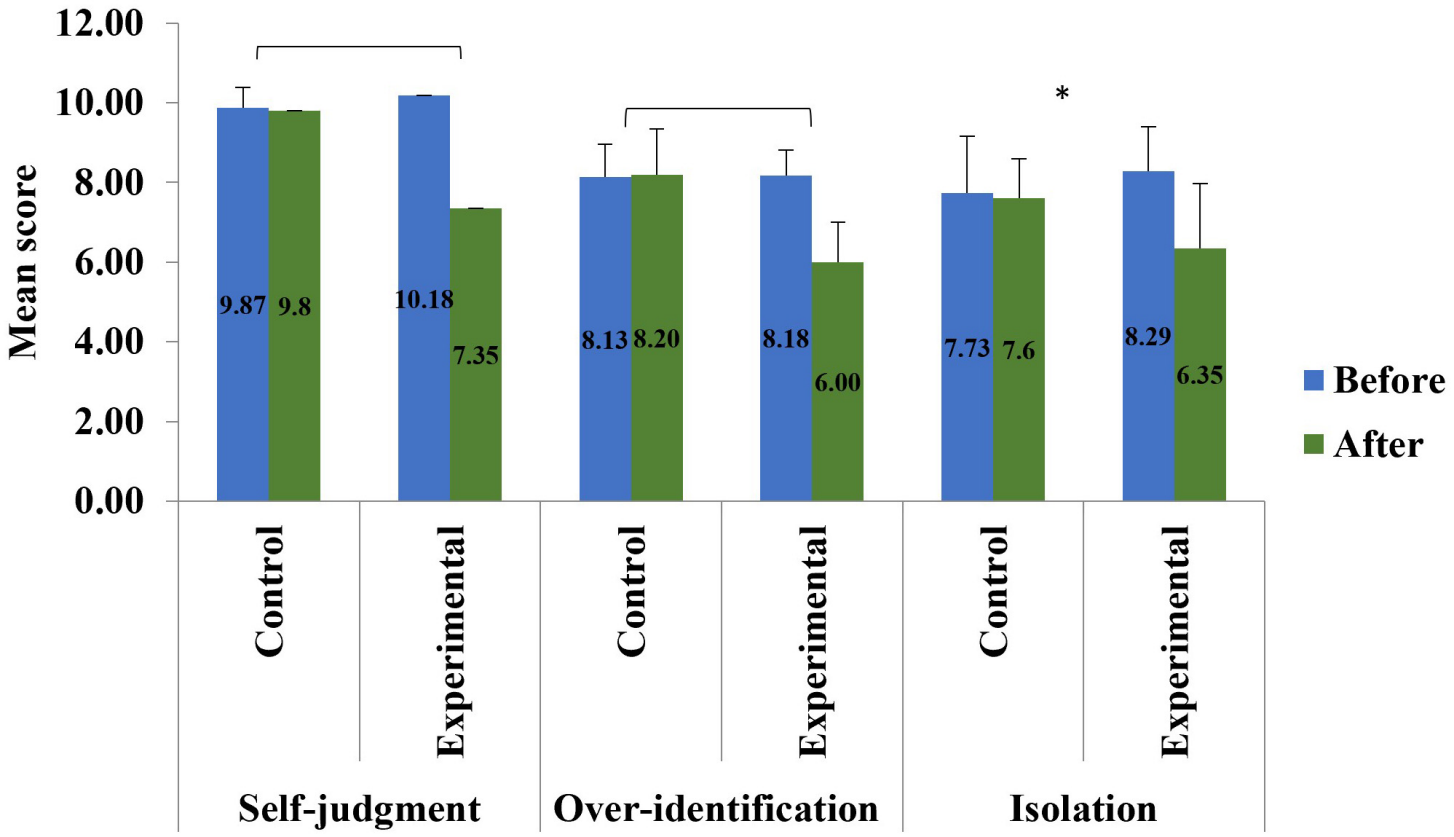
Mean (±SD) self-compassion scale scores for self-judgment, over-identification, and isolation in control and experimental groups at baseline (pre-intervention) and post-intervention. * Significant differences between pre- and post-intervention are indicated by *p* < 0.05.

**Figure 6:**
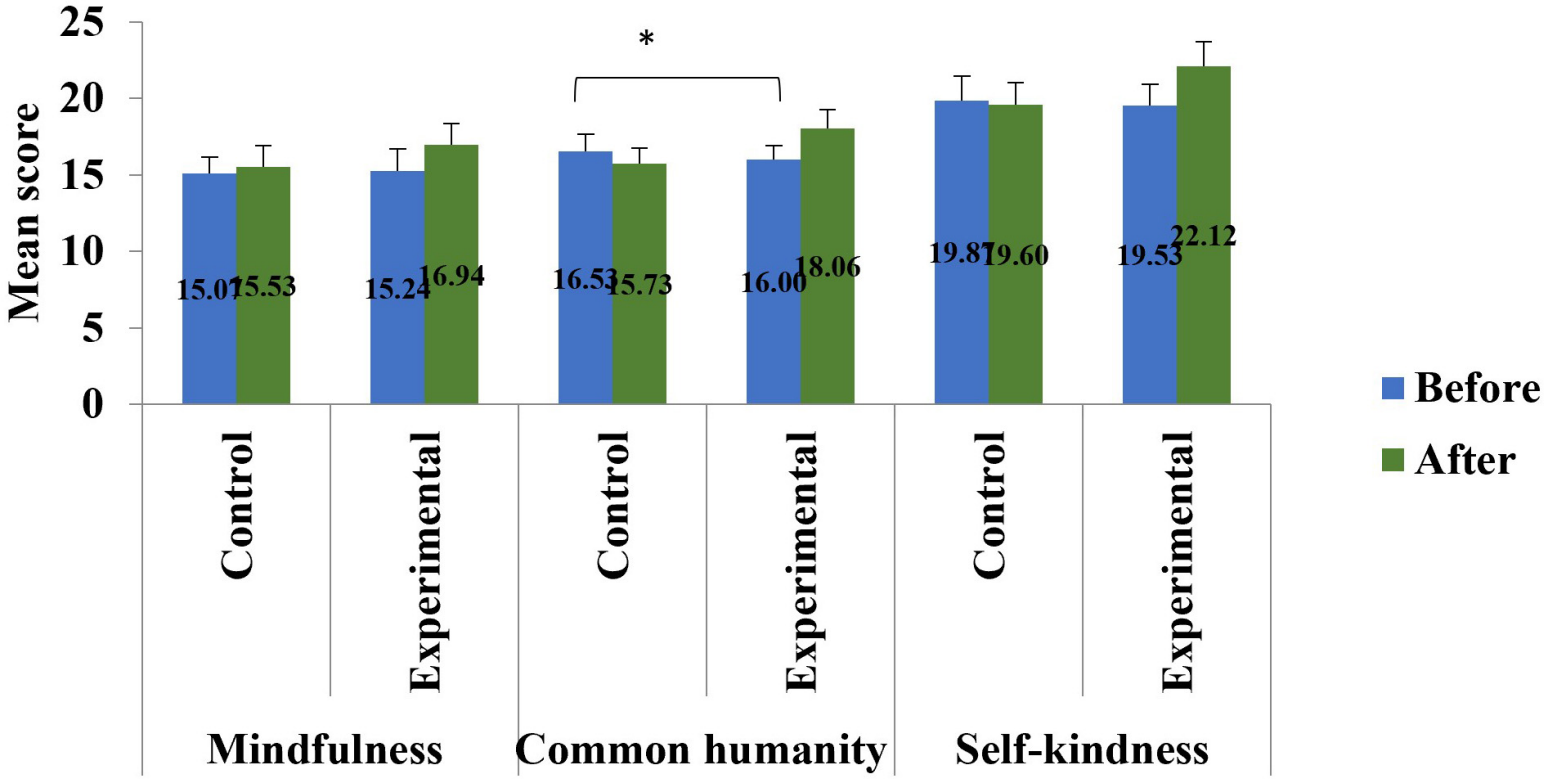
Mean (±SD) self-compassion scale scores for mindfulness, common humanity, and self-kindness in the control and experimental groups at baseline (pre-intervention) and post-intervention. ^*^ Significant differences between pre- and post-intervention are indicated by *p* < 0.05.

### 3.4 Physical performance

Regarding physical performance, results from the 3,000 m cross-country test showed a significant main effect of time (F (1,30) = 4.12, *p* = 0.012, η*p*^2^ = 0.121) and a significant Group × Time interaction (F (1,30) = 7.24, *p* = 0.049, η*p*^2^ = 0.12), indicating greater performance improvements in the experimental group compared with the control group (Figure 7).

**Figure 7:**
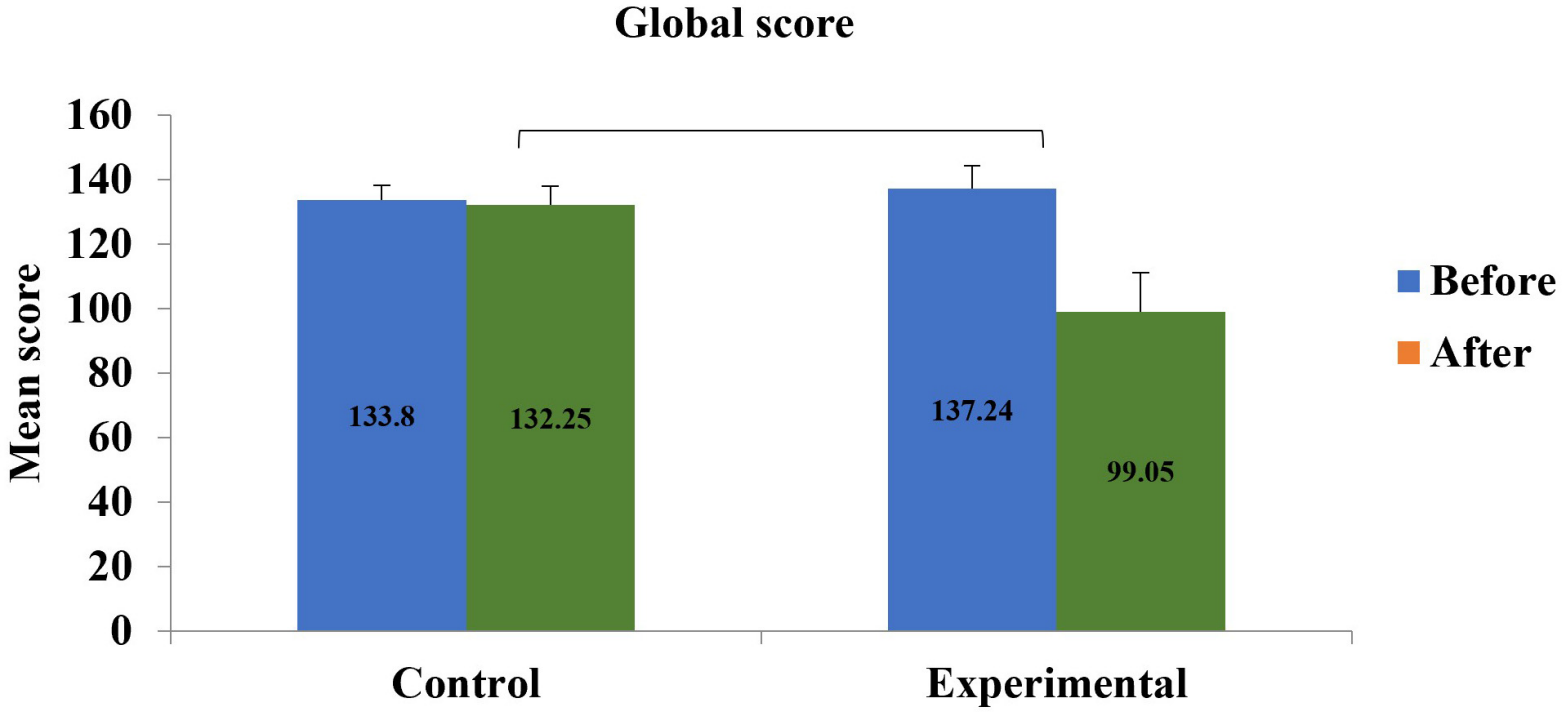
Mean (±SD) 3,000 m performance (min: sec) in the control and experimental groups at baseline (pre-intervention) and post-intervention. ^*^ Significant differences between pre- and post-intervention are indicated by *p* < 0.05.

### 3.5 Between-group differences at post-intervention

After adjustment for baseline scores, the experimental group showed significant improvements relative to the control group across most outcome measures. On the POMS subscales, the experimental group demonstrated significantly lower depression-discouragement (adjusted mean difference = −11.31, 95% CI [−13.98, −8.64], *p* < 0.001), fatigue-inertia (adjusted mean difference = −6.71, 95% CI [−8.60, −4.82], *p* < 0.001), and anger-hostility (adjusted mean difference = −1.93, 95% CI [−3.43, −0.44], *p* = 0.014), alongside significantly higher vigor-activity (adjusted mean difference = 6.31, 95% CI [3.77, 8.85], *p* < 0.001) and interpersonal relationship scores (adjusted mean difference = 6.17, 95% CI [4.15, 8.20], *p* < 0.001). Confusion-perplexity was also significantly higher in the experimental group (adjusted mean difference = 0.83, 95% CI [0.14, 1.51], *p* = 0.020), while anxiety-tension did not differ significantly between groups (*p* = 0.783).

To account for potential baseline imbalances between groups, between-group differences at post-intervention were examined using analysis of covariance (ANCOVA), with baseline scores entered as covariates. Adjusted (estimated marginal) means, 95% confidence intervals, and between-group mean differences are presented in Table 4.

**Table 4:** Adjusted between-group differences in POMS, self-compassion, and performance outcomes at post-intervention (ANCOVA).

Variable	Adjusted mean control [95% CI]	Adjusted mean experimental [95% CI]	Adjusted difference [95% CI]	*p*
Anxiety-tension	31.19 [30.50–31.87]	31.31 [30.63–31.99]	0.13 [−0.84 to 1.10]	0.783
Anger-hostility	27.79 [26.71–28.86]	25.85 [24.82–26.89]	−1.93 [−3.43 to −0.44]	0.014
Confusion-perplexity	21.18 [20.67–21.68]	22.00 [21.55–22.45]	0.83 [0.14–1.51]	0.020
Depression-discouragement	50.86 [48.98–52.74]	39.55 [37.65–41.45]	−11.31 [−13.98 to −8.64]	0.000
Fatigue-inertia	22.29 [20.77–23.80]	15.58 [14.45–16.71]	−6.71 [−8.60 to −4.82]	0.000
Vigor-activity	21.06 [19.18–22.94]	27.37 [25.65–29.09]	6.31 [3.77–8.85]	0.000
Interpersonal relationship	20.26 [18.81–21.72]	26.44 [25.03–27.84]	6.17 [4.15–8.20]	0.000
Self-judgment	9.61 [8.65–10.57]	10.24 [9.62–10.85]	0.63 [−0.51 to 1.77]	0.264
Over-identification	8.23 [7.78–8.67]	8.26 [7.71–8.81]	0.03 [−0.68 to 0.74]	0.932
Isolation	7.90 [7.27–8.52]	8.19 [7.63–8.76]	0.30 [−0.54 to 1.14]	0.468
Mindfulness	15.08 [14.24–15.93]	16.91 [15.94–17.88]	1.83 [0.54–3.11]	0.007
Common humanity	16.39 [15.81–16.96]	17.44 [16.78–18.10]	1.05 [0.17–1.93]	0.021
Self-kindness	19.50 [18.43–20.57]	19.51 [18.49–20.53]	0.008 [−1.47 to 1.49]	0.991
3,000 m performance (min: sec)	9:53 [9:51–9:56]	9:43 [9:40–9:45]	−0:10 [−0:14 to −0:07]	0:006

After adjustment for baseline values, the experimental group showed significantly higher post-intervention scores than the control group for mindfulness (adjusted mean difference = 1.83, 95% CI [0.54, 3.11], *p* = 0.007) and common humanity (adjusted mean difference = 1.05, 95% CI [0.17, 1.93], *p* = 0.021).

No statistically significant between-group differences were observed after adjustment for Self-kindness (mean difference = 0.01, 95% CI [−1.47, 1.49], *p* = 0.991), Self-judgment (mean difference = 0.63, 95% CI [−0.51, 1.77], *p* = 0.264), isolation (mean difference = 0.30, 95% CI [−0.54, 1.14], *p* = 0.468), or Over-identification (mean difference = 0.03, 95% CI [−0.68, 0.74], *p* = 0.932).

Physical performance was associated with a modest improvement in the experimental group, with a slightly shorter adjusted 3,000 m time (adjusted mean difference = −0.10 min, 95% CI [−0.14, −0.07], *p* = 0.006).

## 4 Discussion

The present study aimed to examine the effects of an eight-week self-compassion-based mental preparation program on mood states and endurance performance in cadet cross-country runners. Overall, the findings indicate that the intervention was associated with meaningful improvements in psychological outcomes and modest gains in physical performance. Specifically, significant Group × Time interactions were observed for all mood state variables and self-compassion dimensions, with the experimental group showing greater improvements than the control group. In addition, a significant Group × Time interaction was found for 3,000 m running performance, favoring the experimental group. These results support the study hypotheses and underline the relevance of psychological preparation in youth endurance sport.

First, the findings confirm the importance of psychological preparation in athletic development. Sport psychology literature consistently shows that performance depends not only on physical conditioning but also on psychological skills such as emotional regulation, attentional control, and stress management. Previous research has emphasized that psychological skills training enhances athletes' capacity to cope with competitive demands and maintain optimal functioning under pressure (Vallerand, 2015). Likewise, successful athletes typically demonstrate stronger psychological skills that allow them to regulate emotions and sustain focus in demanding contexts (Krane and Williams, 2006). The improvements observed in the experimental group align with this framework, suggesting that structured mental preparation programs can effectively enhance psychological functioning in young athletes.

Second, the present results extend evidence on self-compassion as a protective psychological resource. According to (Neff 2003a,b), self-compassion involves self-kindness, recognition of common humanity, and mindful awareness of difficult experiences. In sport settings, these processes may help athletes respond more adaptively to mistakes, setbacks, and performance-related stress. The significant reductions in anxiety–tension, anger–hostility, confusion–perplexity, depression–dejection, and fatigue–inertia observed in the experimental group suggest improved emotional regulation and reduced maladaptive self-evaluative processes. These findings are consistent with prior evidence linking higher self-compassion with lower levels of anxiety, depression, and negative affect (Barnard and Curry, 2011; Zhang et al., 2023).

The results are also consistent with broader models of psychological resilience and wellbeing. Resilient individuals are better able to adapt to stress and recover from adversity (Bonanno, 2004), while self-determination theory highlights the importance of psychological resources in supporting optimal functioning and wellbeing (Ryan and Deci, 2000). In this context, self-compassion may act as a buffer against performance-related stress, thereby contributing to the reductions in negative mood states observed in the experimental group.

Another key finding is the significant increase in vigor–activity. Vigor is considered a central indicator of positive mood and readiness to perform. Previous research has shown that optimal athletic performance is often associated with mood profiles characterized by high vigor and low levels of negative affect such as tension, depression, and fatigue (Lane and Terry, 2000; Lane et al., 2012). The observed increase in vigor suggests that self-compassion training may enhance psychological readiness, motivation, and perceived energy in young endurance athletes.

Regarding physical performance, the 3,000 m cross-country results showed a significant Group × Time interaction, indicating greater improvement in the experimental group compared with the control group. Although the absolute performance changes were relatively small (experimental group: 9:55 ± 0:34 to 9:46 ± 0:28 min; control group: 9:48 ± 0:29 to 9:49 ± 0:34 min), even small improvements in middle- and long-distance running can be meaningful in competitive contexts, particularly at the cadet level where performance margins are narrow. Therefore, the statistically significant interaction (*p* = 0.049) may also reflect practically relevant performance gains. The modest magnitude of change may be explained by the relatively short intervention duration and the fact that endurance adaptations often require longer training periods. The observed improvement may be related to psychological factors such as emotional regulation, attentional control, pacing, and fatigue management. However, these mechanisms were not measured in this study and remain speculative, requiring confirmation in future research.

From a practical perspective, these findings support the integration of self-compassion-based strategies into mental preparation programs for young athletes. Developing self-compassion may help athletes manage competitive stress more effectively, reduce maladaptive self-criticism, and maintain a more balanced emotional state during training and competition. For coaches and sport psychologists, incorporating techniques such as guided reflection, mindfulness exercises, and emotional regulation strategies may represent a valuable approach to enhancing both psychological wellbeing and performance development in youth sport.

Despite these promising findings, several limitations should be acknowledged. The design does not allow for the complete isolation of the specific effect of self-compassion from generic group-related factors (social support, novelty of learning, expectancy effects, attentional effects, Hawthorne effect, interaction with facilitators), nor does it fully distinguish it from mindfulness, with which it shares components such as interoceptive awareness. Future studies with an active control group are needed to confirm this specificity. The relatively small sample size and the specific focus on cadet cross-country runners limit the generalizability of the results to other age groups, sports, and competitive levels. The intervention duration of 8 weeks may not have been sufficient to produce larger or more stable physiological adaptations, particularly in endurance performance outcomes. The study relied primarily on self-reported psychological measures, which may be influenced by social desirability or response bias, The sample consisted solely of boys, which prevents generalizing the results to girls participating in cross-country running. Furthermore, the limited age of the participants in the sample (16.0 ± 0.8 years) complicates generalization to the entire adolescent population, as the age limit varies among authors from 12 to 20 years, or even up to 25 years. Future research with composite samples encompassing a wider range of adolescent ages is necessary to validate the universal applicability of these findings. Recruitment within the same club aimed to control extraneous variables (training, coaching, performance level) but limits the generalization of results to other clubs or contexts.

No correction for multiple comparisons was applied across the numerous outcome variables analyzed (POMS subscales, total mood, SCS subscales, and performance); although these outcomes are conceptually and statistically correlated rather than independent, the present findings should be considered preliminary and future studies with adequate statistical power should consider correction for multiple testing (e.g., Holm-Bonferroni or false discovery rate procedures).

Finally, the absence of long-term follow-up limits the ability to determine whether the observed psychological and performance improvements are maintained over time. Future research should address these limitations by including larger and more diverse samples, extending intervention duration, incorporating objective psychological and physiological indicators, and conducting longitudinal follow-up assessments.

## 5 Conclusions

The present study demonstrates that an eight-week self-compassion-based mental preparation program was associated with enhanced psychological wellbeing and a modest improvement in endurance performance in cadet cross-country runners. The intervention led to significant improvements in mood states and self-compassion dimensions, alongside a modest but meaningful improvement in 3,000 m running performance compared with the control group. These findings highlight the value of integrating psychological skills training, particularly self-compassion, into youth sport development programs.

From a practical perspective, self-compassion training represents a simple, low-cost, and adaptable strategy that can be incorporated into regular training routines. Coaches and sport psychologists are encouraged to include structured mental preparation sessions focusing on self-kindness, mindful awareness, and adaptive responses to mistakes and setbacks. Such strategies may help young athletes better manage competitive stress, reduce maladaptive self-criticism, and maintain more stable emotional states during training and competition. Over time, this may contribute not only to improved psychological resilience but also to more consistent performance outcomes in endurance sport contexts.

## Data Availability

The raw data supporting the conclusions of this article will be made available by the authors, without undue reservation.
